# Transcriptional Cascade in the Regulation of Flowering in the Bamboo Orchid *Arundina graminifolia*

**DOI:** 10.3390/biom11060771

**Published:** 2021-05-21

**Authors:** Sagheer Ahmad, Chuqiao Lu, Jieqiu Wu, Yonglu Wei, Jie Gao, Jianpeng Jin, Chuanyuan Zheng, Genfa Zhu, Fengxi Yang

**Affiliations:** Guangdong Key Laboratory of Ornamental Plant Germplasm Innovation and Utilization, Environmental Horticulture Research Institute, Guangdong Academy of Agricultural Sciences, Guangzhou 510640, China; sagheerhortii@gmail.com (S.A.); luchuqiao@gdaas.cn (C.L.); Jieqiu2021520@163.com (J.W.); weiyonglu@gdaas.cn (Y.W.); gaojie@gdaas.cn (J.G.); jinjianpeng@gdaas.cn (J.J.); zcytrain@zhku.edu.cn (C.Z.)

**Keywords:** *Arundina graminifolia*, flowering, transcriptome, transcription factor

## Abstract

Flowering in orchids is the most important horticultural trait regulated by multiple mechanisms. *Arundina graminifolia* flowers throughout the year unlike other orchids with a narrow flowering span. However, little is known of the genetic regulation of this peculiar flowering pattern. This study identifies a number of transcription factor (TF) families in five stages of flower development and four tissue types through RNA-seq transcriptome. About 700 DEGs were annotated to the transcription factor category and classified into 35 TF families, which were involved in multiple signaling pathways. The most abundant TF family was bHLH, followed by MYB and WRKY. Some important members of the bHLH, WRKY, MYB, TCP, and MADS-box families were found to regulate the flowering genes at transcriptional levels. Particularly, the TFs WRKY34 and ERF12 possibly respond to vernalization and photoperiod signaling, MYB108, RR9, VP1, and bHLH49 regulate hormonal balance, and CCA1 may control the circadian pathway. MADS-box TFs including MADS6, 14, 16, AGL5, and SEP may be important regulators of flowering in *A. graminifolia*. Therefore, this study provides a theoretical basis for understanding the molecular mechanism of flowering in *A. graminifolia*.

## 1. Introduction

Flowering is the key developmental stage resulting from vegetative to reproductive transition [1]. Plants have developed several genetic mechanisms to assure flowering in various environmental conditions. These mechanisms are regulated by intrinsic and extrinsic signals [2]. To date, six flowering control pathways have been presented, including the ambient temperature pathway, vernalization pathway, circadian clock pathway, photoperiod pathway, gibberellin pathway, and autonomous pathway [3]. In the vernalization, photoperiod, and ambient temperature pathways, light and temperature act as extrinsic signals and are important environmental factors in the control of flowering [3,4]. Intrinsic signals are received by the circadian pathway, gibberellin pathway, and autonomous pathway to regulate flowering [5,6,7].

In the model angiosperm *Arabidopsis thaliana*, flowering time depends on five genetic pathways: the vernalization, gibberellin, photoperiod, aging, and autonomous pathways [8]. These pathways are integrated by the function of the genes, including *FLOWERING LOCUS T* (*FT*), *SUPPRESSOR OF OVEREXPRESSION OF CO 1* (*SOC1*) and *FLOWERING LOCUS D* (*FD*) [8,9,10]. These integrators transmit the floral induction signals to the floral meristem identity genes *APETALA1* (*AP1*) and *LEAFY* (*LFY*), which initiate the floral morphogenesis program. Then, floral organs develop under the control of MADS-box genes and their co-regulators [10]. In the photoperiod pathway, cryptochrome and phytochrome photoreceptors detect light signals and retune the feedback loop of the circadian clock, including the timing of cab expression 1 (TOC1), CIRCADIAN CLOCK ASSOCIATED 1 (CCA1) and late elongated hypocotyl (LHY) [11,12]. Constans (CO) plays an important role in the biological clock and photoperiod pathways and promotes flowering through the transcription of the flowering locus T (FT) [12,13,14]. MADS-box TF and FLC (flowering locus C) repress flowering by preventing FT and SOC1 (suppressor of overexpression of CO1) [15]. However, the FLC transcription is repressed by VRN2 (vernalization 2) and VRN3 (vernalization 3) in the vernalization pathway to promote flowering [13,14,16,17]. The short vegetative phase (SVP) is another MADS-box TF that plays a role in the ambient temperature pathway. It negatively regulates FT to negate flowering [18]. The autonomous pathway downregulates FLC transcription and promotes flowering [6]. Gibberellin 20 oxidase (GA20ox) is involved in the GA biosynthesis [2]. It accumulates abundantly in the meristem just before floral induction and promotes flowering by upregulating SOC1 [5]. SPL (squamosa promoter binding protein-like) TFs, which are targeted and depressed by miR156 [19], increase in the circadian clock pathway and promote flowering by upregulating flowering genes, such as FRUITFUL (FUL) and SOC1 [2].

In the plant kingdom, flower development is arguably the most intricate developmental process. Most of the major hormones have been proposed to play roles in flower development, including ABA [20], GA [21], auxin [22], cytokinin [23], jasmonate [24], and ethylene [25]. Although the model plants provide a useful source for the identification and functional characterization of flowering genes, studying these genes in other plants growing in peculiar environments or with unique characteristics is vital to further deepen the molecular basis of flowering regulation. *Arundina graminifolia*, commonly called ‘bamboo orchid’, is an important flower in the family Orchidaceae. It mainly grows in tropical and sub-tropical areas in Asia and has been used as a traditional medicinal plant in China and an ornamental flower in Singapore and Malaysia [26,27,28]. Unlike other orchids, it blooms throughout the year with flowering peaks from September to January. However, the molecular mechanism of flowering in *A. graminifolia* remains unclear. Therefore, the present study uses transcriptome data to mine transcription factor families and important TFs involved in flowering in *A. graminifolia*. Specific results from this study will aid the further understanding of genetic regulation of flowering in orchids.

## 2. Materials and Methods

### 2.1. Plant Materials and Growth Conditions

*Arundina graminifolia* plants were grown at the greenhouse of Environmental Horticultural Research Institute of Guangdong Academy of Agricultural Sciences, China (23°9′ N, 113°21′10″ E). Each cluster contained 3 to 4 plants with a cluster distance of 10 cm × 10 cm. Plants were grown at a photoperiod of 16/8 h with a day/night temperature of 25/20 °C. Samples were collected from five stages (stage 1–5) of floral development (FD), flowers, silique, leaves, and root. Sampling was done in three biological and technical repetitions for each floral development stage and tissue type. All the samples were collected in liquid nitrogen and immediately stored at −80 °C for further processing. 

### 2.2. Horticultural and Flowering Characteristics Assessmenet from Different Sources

Horticultural traits and flowering characteristics of A. graminifolia were observed from four different sources, including Guangdong (China), Hainan (China), Singapore, and Malaysia. The data were recorded for plant height, number of blades, leaf length, leaf width, stem diameter, flowering period, and capsule color (Table 1). A total of 20 plants were observed from each source. Leaf characteristics were measured from the strongest and most adult leaves, i.e., the 4th and 5th leaf. Stem diameter was measured from the widest part of stem, i.e., the middle of the stem.

### 2.3. RNA-Seq Library Preparation and Sequencing

RNA was extracted from 9 tissues (5 FD stages, flower, silique, root, and leaf) using a TaKaRa RNA extraction kit, followed by the construction of cDNA libraries. Oligotex mRNA Midi Kit (QIAGEN, Hilden, Germany) was used to filter mRNAs from total RNA. RNA quantity and quality was checked on a Nano-Dropt 2000 spectrophotometer (Thermo Scientific, Waltham, MA, USA), followed by cDNA library preparation using Illumina manufacturing protocol. The mRNAs were fragmented to an approximate length of 200 bp. The first and second strand cDNA was synthesized from the isolated mRNAs, followed by adapter ligation and low-cycle enrichment according to TruSeq^®^RNA HT Sample Prep Kit (Illumina, San Diego, CA, USA). The purified library products were evaluated using Agilent 2200 TapeStation and Qubit^®^2.0 (Life Technologies, Waltham, MA, USA) and diluted to 10 pM to generate clusters in situ on the HiSeq2500 pair-end flow cell. Sequencing (2 × 100 bp) was performed. Each sample produced about 60 million reads. Transcriptome *de novo* was done using the Trinity program with default parameters [29].

### 2.4. Analysis of DEGs

Gene expression was calculated by RPKM values using the following formula:

RPKM = [total exon reads/mapped reads (millions)] × exon length (kb)

The edgeR package was used to determine the significant difference among tissues. The threshold *p*-value was determined using the false discovery rate (FDR) at FDR <0.05 and |log2 ratio| >1 (two-fold change). We screened the DEGs with an FDR threshold of 0.05 or less and the log2 ratio of one or more. The DEGs were annotated to the gene ontology (GO) and Kyoto Encyclopedia of Genes and Genomes (KEGG) databases (http://www.geneontology.org/or http://www.genome.ad.jp/; accessed on 5 November 2018) to calculate gene enrichment for biological processes or KEGG pathways. Genetically enriched DEGs were found using a hypergeometric test. Significantly enriched GO terms or KEGG pathways were filtered at a *p* or *q* value of ≤0.05. 

### 2.5. GO Biological Process Enrichment for Flowering

Of the 25,353 DEGs, about 1000 DEGs were filtered according to flower development, flowering time regulation, circadian clock, and hormonal regulation. The annotated gene IDs were run on ShinyGO (Gene Ontology Enrichment Analysis; v0.61) to perform network analysis and connection tree analysis. 

### 2.6. Filtering of TF Families

The term transcription factor was searched across DEG annotations and sorted 687 terms labeled as transcription factors. The filtered terms were sorted to find the subsets of major TF families. 

### 2.7. Identification of Significantly Differential and Stage Specific Tfs

Top 20 highly differential TFs were identified across all the samples using the edgeR package of R.

Preferentially expressed/stage specific genes were mined using a stage specificity (SS) scoring algorithm. This algorithm compares the gene expression in a specific stage with its maximum expression in other stages [30]. The higher SS score of a gene in a particular stage signifies its expression at that stage. SS scores were used to draw a heatmap using ggplot2 utility in R.

### 2.8. Quantitative Real-Time PCR Analysis

Highly expressed stage specific genes were selected for validation through qRT-PCR. RNA was extracted from 9 tissues and cDNA was obtained using the protocol by Fermentas. The qRT-PCR was performed in a reaction mixture of 20 µL containing 10 µL of SYBR premix Ex-taq™ (Takara, Kusatsu, Japan) using Bio-Rad CFX-96 RealTime PCR System (Bio-Rad, Hercules, California, USA). *Actin* was used for the internal standardization. Three biological and technical repeats were performed for each sample. 

### 2.9. Statistical Analysis

ANOVA (one-way) was used in SPSS software (SPSS Inc., Chicago, IL, USA; ver. 16.0) to judge the statistical significance of data. Significant differences are shown at *p* < 0.05 or *p* < 0.01 level.

## 3. Results

### 3.1. Flowering Habit of A. graminifolia

*A. graminifolia* starts the reproductive cycle after six months of vegetative growth. This time span is very short compared to other orchids, such as *Cymbidium* and *Phalaenopsis*, taking 2–3 years to complete the juvenile phase. Moreover, it flowers throughout the year, with flowering peaks between September and January (Table 1). The average life of a single flower is about one month. The flower development is completed in five stages, starting from stage 1 to the fully formed flower (stage 5). The mature flower bears a column in the center with four pollinia arranged in a semi-circle around it (Supplementary Figure S1). To obtain a comprehensive understanding of the genetic regulation of flower development, we performed transcriptome analysis from five stages of floral development, mature flowers, silique, root, and leaves.

### 3.2. RNA-Seq and Functional Annotation

The transcriptome analysis produced about 71.2 billion reads from five development stages and four tissue types. Each sample generated about 10.8–12.8 Gb data with an average of 7.8 billion reads. We filtered 25,353 differentially expressed unigenes (DEGs), which were annotated using GO, KEGG, Pfam, eggNOG, and NR databases, covering annotation data of 67.73%, 36.42%, 64.18%, 56.54%, 74.37%, and 72.29%, respectively (Supplementary Table S1). 

### 3.3. Biological Process Enrichment of Hormonal and Flowering Related DEGs

**Figure 1:**
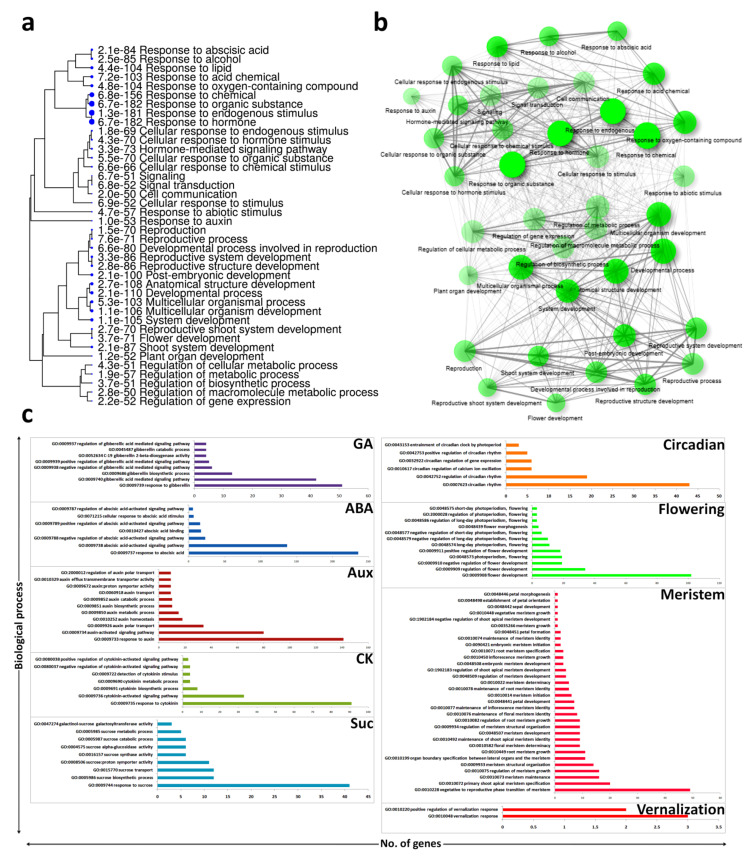
GO (Gene Ontology) biological process annotation; (**a**) interconnections of important abundant biological processes, (**b**) clustering of biological processes of flowering and hormonal related DEGs, (**c**) GO ids of biological processes specific to hormones and flowering regulatory pathways and meristem.

About 1000 DEGs were filtered according to flower development, flowering time regulation, circadian clock, and hormonal regulation. The biological process enrichments of these DEGs show some clusters related to hormonal regulation and flowering (Figure 1a,b). In gibberellic acid regulation, GA-mediated signaling pathway (GO: 0009740) and response to gibberellin (GO: 0009739) were the most abundant biological process terms enriched by 43 and 52 DEGs, respectively. In the abscisic acid regulation, the ABA-activated signaling pathway (GO: 0009738) and response to ABA (GO: 0009737) were the most abundant biological processes enriched by 138 and 237 DEGs, respectively. Auxin transport (GO: 0060918), Auxin biosynthetic process (GO: 0009851), auxin homeostasis (GO: 0010252), auxin polar transport (GO: 0009926, auxin activated signaling pathway (GO: 0009734), and response to auxin (GO: 0009733) were shown by 9, 10, 18, 34, and 141 DEGs, respectively. The cytokinin biosynthetic process (GO: 0009691), cytokinin-activated signaling pathway (GO: 0009736), and response to cytokinin (0009735) were regulated by 8, 33, and 91 DEGs, respectively. Response to sucrose (GO: 0009744), sucrose biosynthetic process (GO: 0005986), and sucrose transport (GO: 0015770) were the abundant processes in the sucrose regulation regulated by 42, 12, and 12 DEGs, respectively (Figure 1c).

The circadian rhythm (GO: 0007623) and the regulation of the circadian rhythm (GO: 0042752) were the most abundant biological processes in the circadian regulation regulated by 44 and 19 DEGs, respectively. A few DEGs were involved in the vernalization response (GO: 0010048). The DEGs enriched in the long day photoperiodism and flowering (GO: 0048574), negative regulation of flower development (GO: 0009910), photoperiodism and flowering (GO: 0048573), positive regulation of flower development (GO: 0009911), regulation of flower development (GO: 000909), and flower development (GO: 0009908) were 11, 19, 19, 35, and 102, respectively. In the organ specification, vegetative to reproductive phase transition of meristem (GO: 0010228), petal development (GO: 0048441), regulation of meristem growth (0010075), primary shoot apical meristem specification (0010072), floral meristem determinacy (GO: 0010852), maintenance of flora meristem identity (GO: 0010076), and maintenance of inflorescence meristem identity (GO: 0010077) were the important biological processes regulated by 50, 7, 16, 21, 9, 8, and 7 DEGs, respectively (Figure 1c).

### 3.4. Filtering of Transcription Factor (TF) Families

**Figure 2:**
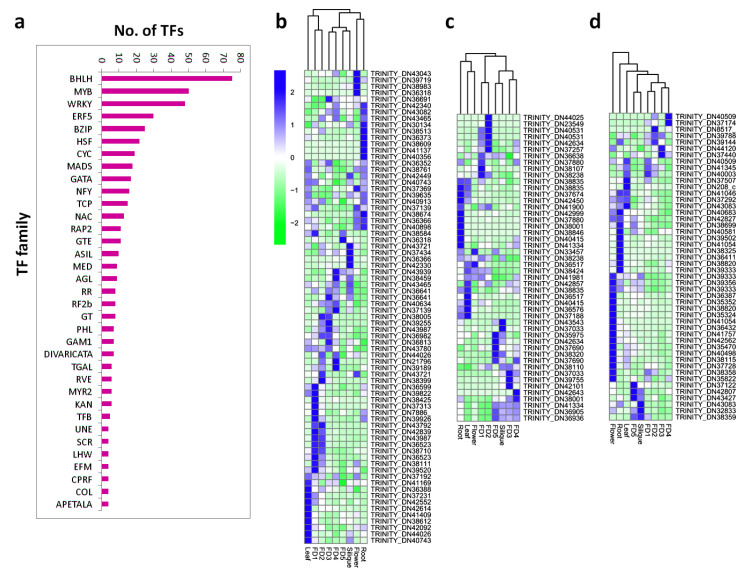
Abundance of TF families; (**a**) number of TFs in all major families, (**b**) bHLH TF family, (**c**) MYB TF family, (**d**) WRKY TF family.

The term ‘Transcription Factor’ was searched across annotations to find DEGs related to transcriptional activity. We found about 700 DEGs involving transcriptional activity, which were divided into 35 different families. A number of famous families were found in our transcriptome data, including bHLH, MYB, WRKY, EFF, bZIP, CYC, MADS, TCP, and NAC (Figure 2a). The bHLH was the most abundant TF family, containing more than 70 members (Figure 2b), followed by MYB, containing about 50 members (Figure 2c), and WRKY, containing more than 45 members (Figure 2d). The bHLHs were mainly expressed in the root, leaf, and early stages of flower development, especially in FD1. The MYBs were also expressed in the root, leaf, and early stages of flower development. However, the WRKYs were expressed abundantly in the flower and root compared to other floral development stages or tissue types.

### 3.5. Tissue-Specific Up and Down Regulation of TFs

**Figure 3:**
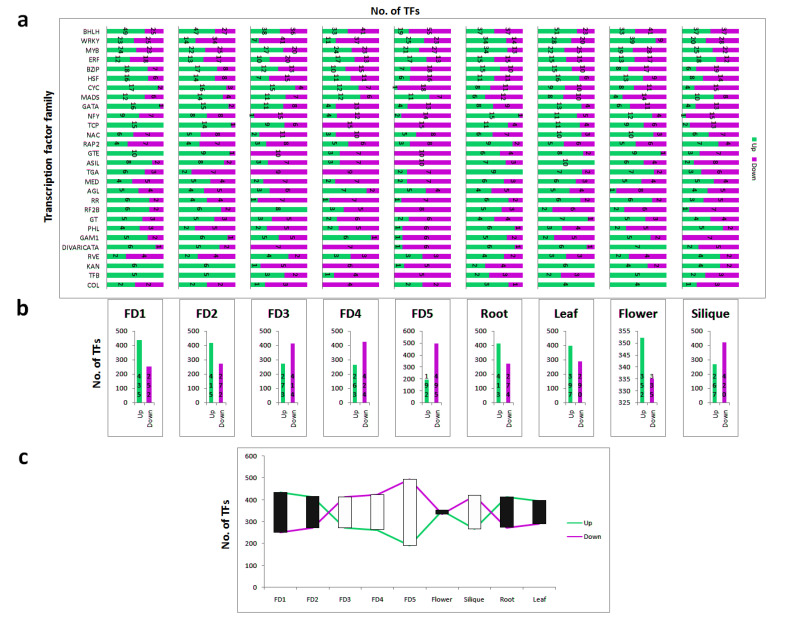
(**a**) Number of up- and downregulated TFs, (**b**) stage specific number of up- and downregulated TFs, (**c**) relation of up- and downregulation across different tissues.

Interesting relationships were found among TF families across developmental stages and tissue types. As shown in Figure 3a, the ratio of up- and downregulated TFs varies across all the tissues. Among the major TF families, bHLH and ERF (ETHYLENE RESPONSE FACTOR) did not vary too much across developmental stages or tissue types. However, WRKY showed great variations in FD3, FD4, and flower; downregulated in FD3 and FD4, while upregulated in flower. CYC TFs were gradually downregulated from FD1 to FD4 and showed complete downregulation in FD5. MADS were mainly upregulated in the flower development stages, while most of them were downregulated in the roots. Similarly, COLs were also upregulated during flower development and remained low in non-reproductive parts (Figure 3a).

Comparing the floral development stages (Figure 3b), FD1 showed the most upregulated TFs (435), whereas FD5 showed the most downregulated TFs (495). A reciprocal trend can be seen between upregulated and downregulated TFs across five stages of flower development (Figure 3b). Upregulation showed a decreasing trend from FD1 to FD5, whereas downregulation showed an increasing trend from FD1 to FD5. The maximum difference of up- and downregulated TFs was observed in FD5, wherein 495 TFs showed upregulation and 192 TFs shown downregulation. However, in the mature flower an equal number of up- and downregulated TFs can be seen (Figure 3c). These results suggest that the gene expression profiles of the samples obtained at the early and late flowering stages are quite different, and many genes may be highly expressed at the initial stage of flowering (FD5), and their expression may decrease as the flowers bloom and with age.

### 3.6. Top 20 Highly Differential TFs

**Figure 4:**
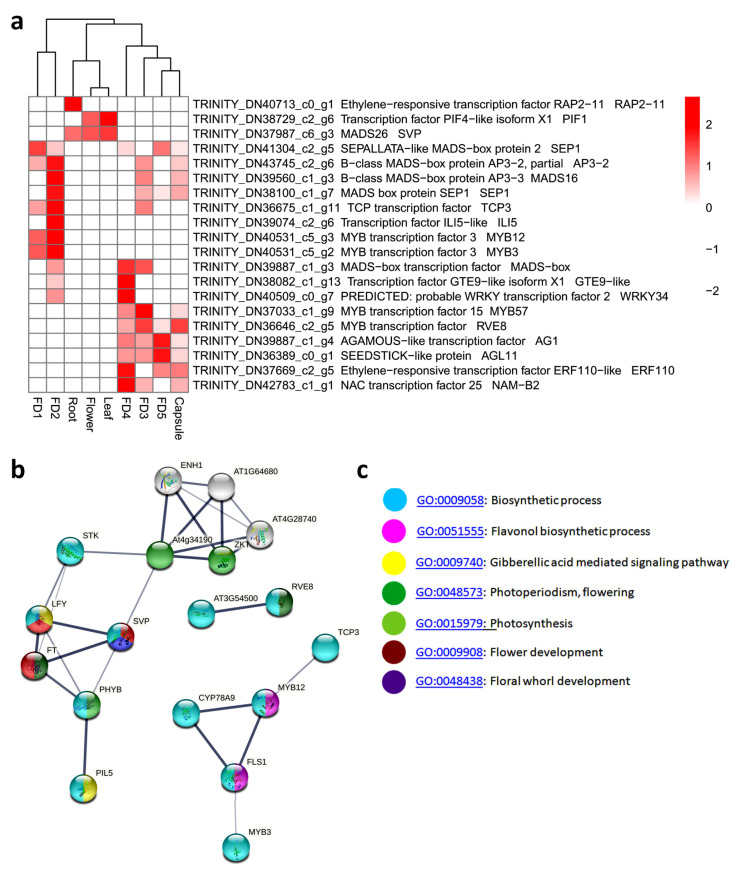
Top 20 most differential TFs; (**a**) heatmap of top 20 TFs, (**b**) string-based protein–protein interaction of some candidates of top 20 TFs, (**c**) enrichment of important biological process terms related to flowering and hormones in top 20 TFs shown in part ‘**b**’.

The edgeR package of R was used to filter the top 20 highly differential TFs across nine tissues. These include some of the known regulators of flowering reported in model plants and other orchid species. Notably, this highly differential set of genes contained eight MADS-box TFs and three MYBs. Cluster analysis of these 20 DEGs showed that FD1 and FD2 in early flower developmental stages were clustered together, while FD3, FD4, and FD5 in the late stages were clustered together. The former mainly showed upregulated expression of class E MADS gene *SEP*, class B MADS gene *AP3*, *TCP,* and two *MYBs*, while the latter showed a higher expression of class C MADS gene *AG-like* and class D MADS gene *STK* (*SEEDSTICK*), as well as *WRKY* and *EFR* genes. In vegetative organs, the accumulation of the flowering suppressor genes *SVP* and *PIF1* (*Phytochrome-interacting factor*) was higher. This indicated a close relation between these genes with floral organ development. By analyzing the interaction proteins of these key genes, we found many other important factors linked together, for example, SVP is linked with leafy (LFY) and FT. Moreover, MYB12 involves the chemical homeostasis along with TCP3 (Figure 4b). Important biological processes were observed in the top 20 highly differential TFs, such as flower development (GO: 0009908), flower whorl development (GO: 0048438), and photoperiodism and flowering (GO: 0048573) (Figure 4c). Most of the highly differential DEGs identified in *A. graminifolia* are similar to the genes identified in other orchids, such as *Dendrobium catenatum*, *Phalaenopsis equestris*, *Cymbidium goeringii*, and *Cymbidium sinense* (Supplementary Table S3).

### 3.7. Stage-Specific Highly Upregulated TFs

**Figure 5:**
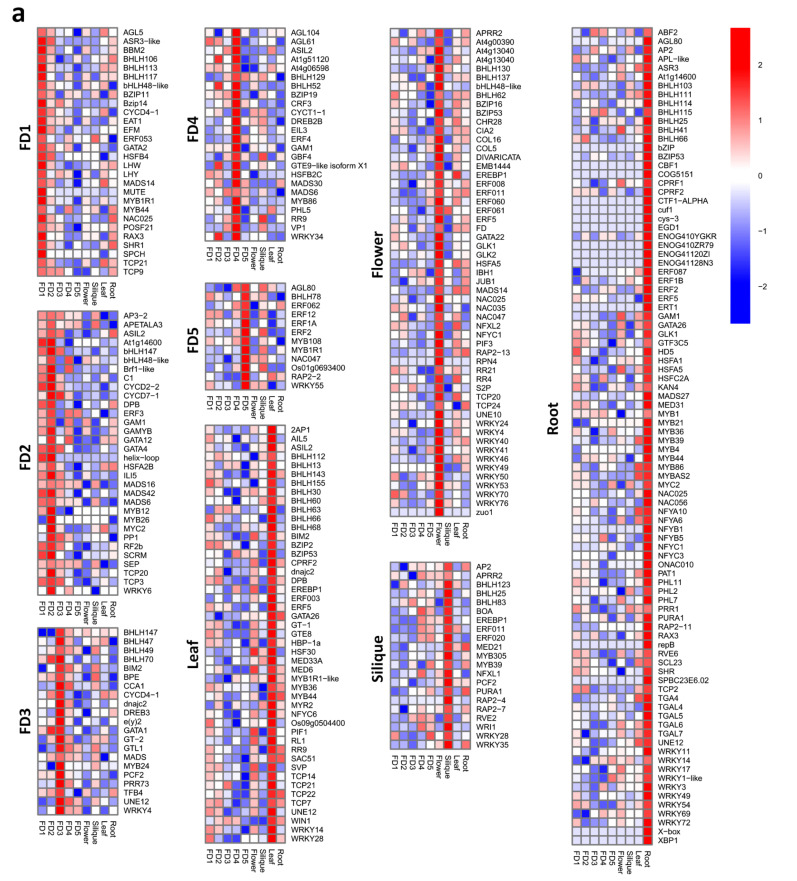

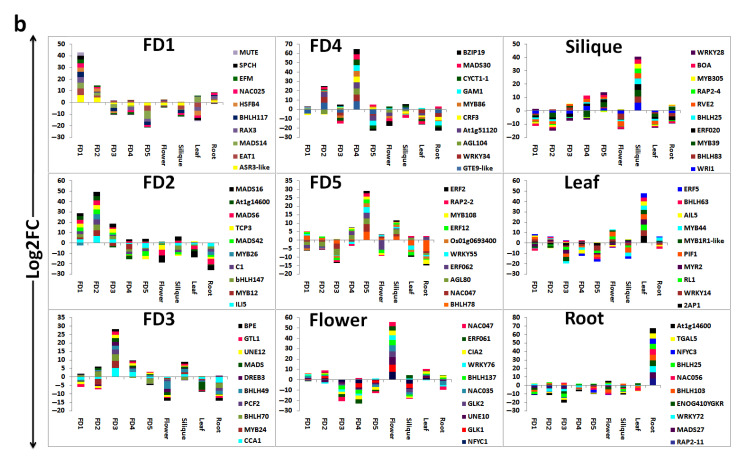
Stage-specific TFs; (**a**) heatmap of stage specific TFs, (**b**) log2-foldchange expression trends of top 10 stage-specific TFs.

Stage-specific TFs were identified to know the specific response of each stage and tissue type. The minimum number of TFs (12) was expressed specifically in FD5, whereas the maximum number of TFs (92) was specific to the root (Figure 5a). Regarding the floral development, 32 TFs showed the highest expression in FD2, followed by FD1 (28) and FD4 (24). Generally, the differentially expressed genes were similar in the two adjacent developmental stages. Most of the TFs upregulated in FD1 were also upregulated, although less intensely, in FD2. Similarly the FD3-specific TFs were also upregulated in less intensity in FD4. By contrast, the expression profile of the root was the most specific. Most of the genes were highly expressed here and downregulated in other tissues.

To obtain a better understanding of the stage-specificity, the top 10 highly expressed TFs were sketched together to elaborate their expression patterns across tissues (Figure 5b). FD1 showed upregulation in multiple TF families, including MADS14, bHLH117, and *ETERNAL TAPETUM1* (*EAT1*) with their roles in flower development. Only one of the FD1 TFs was downregulated in FD2, and they were completely downregulated from FD3-FD5. 

The TFs showing the highest expression in FD2 were also upregulated in FD1. They were gradually downregulated in other stages of flower development and tissue types. In FD1, two flowering-related TFs MADS14 and EAT1 showed the highest expressions. MADS14 is 88% identical to FRUITFUL-like MADS protein in *Cymbidium goeringii* (accession no. KX347442.1). EAT1 is similar to *Phalaenopsis equestris* EAT1 TF (88% similarity; accession no. XM_020741375.1). Interestingly, FD2 showed the highest expression of some famous regulators of flowering, such as MADS6, MADS16, TCP3, and MYB12. MADS6 and MADS16 are 91% and 90% similar, respectively, to *Dendrobium catenatum* MADS6 (accession no. XM_020843535.2), and MADS16 (accession no. XM_020822071.2). In FD3, the upregulated TFs were more specific compared to FD1 and FD2. CCA1 is the important circadian clock regulator of flower timing upregulated in this stage. It is similar (87%) to *Dendrobium catenatum* CCA1 protein (accession no. XM_020842163.2).

The expression trend of FD4- and FD5-specific TFs was very different compared to rest of the stages of flower development. WRKY34 is specifically expressed in FD4. It is similar (76%) to *Gossypium raimondii* TF WRKY34 (accession no. XM_012605143.1). In FD5, AGL80 (86% similar with *Phalaenopsis equestris* agamous-like MADS-box protein AGL80; accession no. XM_020720626.1), and ERF12 (91% similar with *Musa acuminata* subsp. ethylene-responsive transcription factor 12-like; accession no. XM_009398926.2) were the notable flowering related TFs (Supplementary Table S3). Interestingly, when comparing the reproductive parts of *A. graminifolia*, the TFs upregulated in the mature flower were mostly downregulated in other reproductive parts. This indicates that the expression of these genes increases at the end of the flowering process, which may be related to the flower senescence. 

TFs in the root showed the maximum expression values, wherein the cumulative log2 fold change was greater than 70 compared to the rest of the tissues. Furthermore, the TFs expressed here were completely downregulated in all other plant parts or floral development stages. Similarly, the leaf also showed distinct TFs, which mostly downregulated in other tissues, except for the flower and FD1, where only a few TFs showed upregulation with negligible intensities (Figure 5b).

### 3.8. The qRT-PCR Validation of Some Selected TFs in Flower Regulation

**Figure 6:**
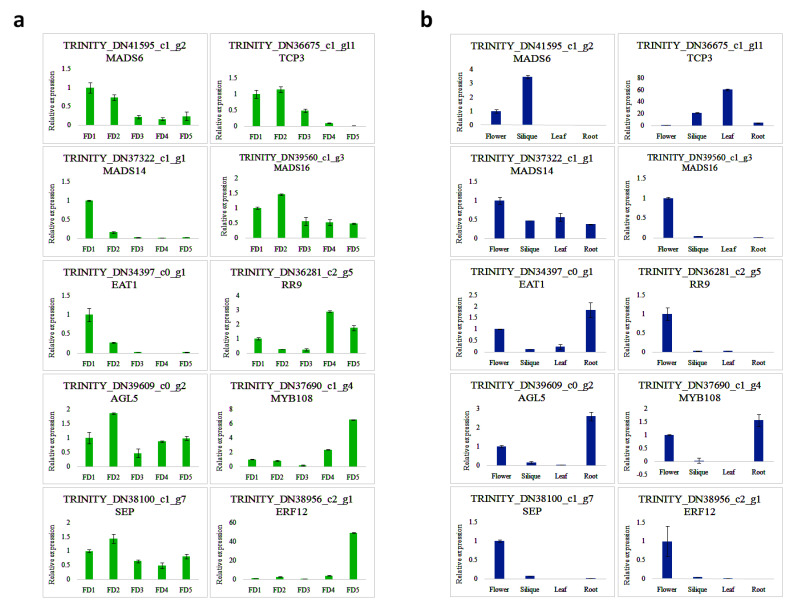
qRT-PCR validation of selected TFs; (**a**) qRT-PCR expression of selected TFs in five stages of flower development, (**b**) qRT-PCR expression of selected TFs in other tissue types.

The expression trends of ten randomly selected TFs were ascertained (Figure 6). The results from quantitative PCR showed that the expressions of most of the genes were consistent with that of transcriptomic expressions (Supplementary Figure S2). For example, *MADS6*, *MADS14*, and *EAT1* showed the highest expressions in FD1 as compared to other stages of floral development (Figure 6a). However, in other tissues, the expression was the highest in silique compared to the mature flower, leaf, and root, where it was negligible (Figure 6b). In FD2, *TCP3*, *SEP*, and *MADS16*, showed the highest expressions; while in other tissues, their expression was the highest in silique. The maximum expression of *RR9* was observed in FD4 among all stages of floral development and it was also abundant in silique and mature flowers. Regarding FD5, *MYB108* and *ERF12* were the most abundant compared to the rest of the floral development stages. However, in other tissue types, *MYB108* showed high expression in the root, silique, and mature flower, while *ERF12* was abundant in the silique.

## 4. Discussion

Currently, transcriptome has been widely used to identify genes regulating unique plant traits [31,32,33,34]. In our study, 25,353 unigenes were assembled from five floral development stages and four tissue types of *A. graminifolia*. Using the DEGs as reference, 687 transcription factors were identified in the floral organs and other tissues. Flower development in *A. graminifolia* may be coregulated by a variety of TFs. Our findings, thus, provide the genetic information for the functional characterization of these TFs in orchid flower development. 

More than 35 TF families were identified that may play a role in the regulation of flower development in *A. graminifolia*. Our data suggested some important regulators of flower development, hormones, and flowering time that have been previously identified in other orchids, such as *Dendrobium catenatum*, *Phalaenopsis equestris*, *Cymbidium goeringii*, and *Cymbidium sinense* (Supplementary Table S3). *HECATE 3* (*HEC3*) regulates phytochrome signaling in the light pathway [35], while one CCA1, 2 LHY, and 4 CO-like TFs were identified from the DEGs in this study. Together with *HEC3*, they may regulate the circadian clock and photoperiod pathways in the flower development of *A. graminifolia*. Gibberellins (GA) are the endogenous signaling molecules that are involved in flowering control. Multiple TFs are known to respond to GA, while ERF and bHLH are involved in ethylene and auxin signaling, respectively [2]. Ethylene and auxin may act antagonistically or synergistically with GA during the flowering regulation [36,37,38,39]. The Viviparous-1 (VP1) encodes a B3 type TF and works during ABA signaling pathway [40]. ABA-INSENSITIVE3 (ABI3) is the ortholog of VP1 in Arabidopsis [41]. Thus, our transcriptomic data generated TF regulators of major hormones involved in flowering regulation. 

Four families of TFs, ZFP, WRKY, bHLH, and MYB have been known to involve flowering regulation in many plant species [2]. In Arabidopsis, some members of bHLH, MYB, and WRKY families, such as bHLH48 and 60; EARLY FLOWERING MYB PROTEIN (EFM); WRKY12, 13, and 71 have been documented to regulate flowering through the transcription of FLOWERING LOCUS T (FT) [33,42,43,44]. MYB, bHLH, and WRKY were the most abundant TF families in our transcriptomic data. Moreover, important MADS-box TFs, including MADS6 (agamous-like 6, AGL6), AGL5, MADS14, MADS16, sepallata (SEP), and apetala3 were identified in the present study. AGL6 and SEP3 play pivotal roles in the regulation of flowering time and floral organ development [45,46]. 

Our data suggested a rich number of TFs that expressed significantly to particular stages of flower development, including FD1 to FD5. MADS14 is an APETALA1 (AP1)/FRUITFUL (FUL)-like MADS-box TF involved in floral meristem identity [47,48,49]. It was induced in shoot apical meristem (SAM) during transition to reproductive growth in *Oryza sativa* [50]. Our qRT-PCR result showed that *MADS-14* was highly expressed in FD1 as compared to other stages of flower development, proposing its important role in meristem identity. Another MADS-box TF *SEP* showed the highest expression in FD2, while *MADS6* showed higher expressions in FD1 and FD2 compared to other stages. *MADS16*, an AP3-PI subfamily gene, was observed in *A. graminifolia* among all the stages of floral development. These results indicated spatiotemporal expression patterns of MADS-box TFs along with flower development. TCP3 functions as a transcriptional activator of *CO* [51] and it showed the highest expression in FD2 which is, according to the transcriptomic expression (Supplementary Figure S2a). CCA1 (CIRCADIAN CLOCK ASSOCIATED1) is a circadian clock component associated with flowering time. It represses *SOC1* and *GI* through direct interaction with their promoters, implying a connection between the flowering pathways and the circadian clock [52]. It was highly expressed in FD3, as shown by transcriptomic data (Figure 5, FD3).

Arabidopsis MYB108 regulates jasmonate-mediated stamen maturation by acting together with MYB24 [53]. MYB108 plays an important role in the correct timing for anther dehiscence, and in combination with MYB24, it regulates filament elongation, pollen viability and anther dehiscence; moreover, its expression is regulated by upstream TF MYB21 [53]. MYB108 was highly expressed in stage 5 of flower development of *A. graminifolia* (Figure 6a), as suggested by qRT-PCR and transcriptomic data (Supplementary Figure S2a). *ETERNAL TAPETUM1* (*EAT1*) is a bHLH TF involved in tapetal cell-fate decision [54]. The highest expression of *EAT1* was observed in FD1. BHLH49 is an auxin regulator during embryo identity [55]. RR9 is a member of type-B response regulators (RRs) and plays important role in cytokinin signaling [56]. RR9 showed the highest expression in FD4, which is according to its transcriptomic expression (Supplementary Figure S2a). WRKY34 induces proteolysis of FRI to regulate flowering in the vernalization pathway [57]. ETHYLENE RESPONSE FACTOR12 (ERF12), in *Arabidopsis*, is an ortholog of MULTIFLORET SPIKELET1 in rice and it pleiotropically affects floral phyllotaxy, meristem identity, and organ initiation in plants [58]. Dependent on the photoperiod, ERF12 is a putative transcriptional repressor and integrates into the AP2 (APETALA2) pathway to regulate floral organ identity and floral timing [58,59]. In line with the transcriptomic data, ERF12 was abundantly expressed in FD5 compared to other stages, where its expression was negligible (Figure 6a). The expression of TFs validated through qRT-PCR was almost similar to the transcriptomic expression for stages of flower development (Supplementary Figure S2). However, for other tissue types, some TFs showed different expression intensities compared to transcriptomic expression.

The regulatory mechanisms for continuous flowering are intimately linked to dormancy and bud release, but the basic molecular mechanisms are not well elucidated. However, transcriptomic and physiological analyses indicate increased expression of FT/FD and GA biosynthesis-related genes, which promote growth, and concomitant downregulation of ABA pathway genes transiently release dormancy to facilitate bud break [60,61,62]. However, ABA can counter this effect in two ways: it regulates GA levels by inhibiting SVP during short-day photoperiod and downregulates FT/FD at low temperatures.

SHORT VEGETATIVE PHASE (SVP), a flowering-time regulator, positively regulates TCP18 [TEOSINTE BRANCHED, CYCLOIDEA, PCR (TCP)], a mediator of temperature-dependent bud break [63]. Both proteins function in a temperature-sensitive transcriptional module that mediates bud break. *Cymbidium goeringii* SVP2 interacts with CgAP1 and CgSOC1, similar to reports in *Arabidopsis*, *Antirrhinum*, petunia, and rice, which forms the basis of MADS-box TF function [64]. AP1 may serve as a hub between the interacting proteins of the flower induction pathway (such as SOC1 and SVP) and the floral organ identity proteins [45]. In temperature‒ and photoperiod‒dependent flowering pathways, *SVP* interacts with *FLC* and *FLM* (*FLOWERING LOCUS M*), causing *FT* repression and flowering [65,66,67]. Moreover, the GA and ABA hormonal pathway genes are targeted by *SVP* in bud break [68]. In this work, two homologs of *Arabidopsis thaliana SVP* were identified in the transcriptome and both showed highest expression levels in leaves. It was almost undetectable in the five stages of *A. graminifolia* flower development, unlike the expression patterns of *Cymbidium goeringii SVPs* that were trapped in the early stage of flower development (Figure 5). Among the TCP homologs identified, TCP3 exhibited the highest expression levels in the first three stages of floral development. TCP21 was expressed in FD1 and leaves.

**Figure 7:**
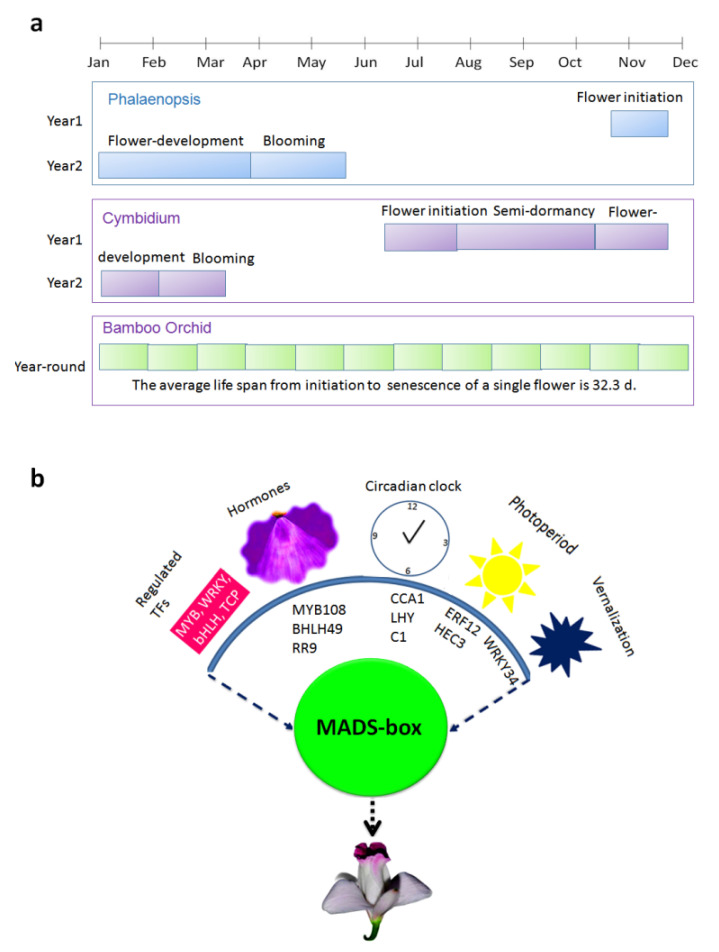
Comparison of flowering pattern of bamboo orchid with Phalaenopsis and Cymbidium (**a**) and the hypothetical model of the flowering regulation involving key transcriptional factors in *Arundina graminifolia* (**b**).

Considering that the regulatory mechanisms for continuous flowering are intimately linked to dormancy and bud release, the most important characteristic of *Arundina graminifolia* is that the initiation of flowering and the floral development are completed rapidly, without going through a dormancy period. Unlike other orchids with narrow flowering span, *A. graminifolia* flowers all year round (Figure 7a). Therefore, studying molecular basis of continuous flowering is of significant interest. Our data show multiple regulatory routes for flowering regulation in *A. graminifolia* (Figure 7b). We found candidate TFs from multiple pathways: vernalization, circadian clock, photoperiod, and hormonal pathways. These pathways interact with floral integrators (MADS-box) to regulate flowering. FT/FD could act as a central receiver of signals to control the flowering time through APETALA (AP1, AP2 and AP3) and SOC1. Moreover, the rapid decrease of *SVP* genes from the early stage may be an important promoting factor to ensure the rapid development of flower organs without dormancy. However, extended research would be needed to reveal these assumptions.

## 5. Conclusions

With the above in mind, we identified a large number of TFs through RNA-seq, which may contribute important roles in different regulatory pathways of *A. graminifolia* flowering. These pathways include the photoperiod, vernalization, hormone, and circadian clock pathways (Figure 7b). The identification of key TFs involved in these pathways can do a great deal to reveal the genetic regulatory network that drives continuous flowering specifically in *A. graminifolia*, and seasonal flowering in other orchids, in general. However, further studies are required to fully unravel the functions of these transcription factors.

## Figures and Tables

**Table 1:** Horticultural characters and flowering characteristics of *A. graminifolia* from different sources.

Origin	Plant Height (cm)	Number of Blades (cm)	Leaf Length (cm)	Leaf Width (cm)	Stem Diameter (cm)	Flowering Period	Silique
Guangdong, China	36.43 ± 1.53	18.74 ± 0.75	10.56 ± 0.48	0.81 ± 0.03	0.32 ± 0.02	Throughout the year	Purple
Hainan, China	31.09 ± 1.42	18.33 ± 0.84	11.59 ± 0.53	0.82 ± 0.03	0.31 ± 0.01	Throughout the year	Green
Singapore	80.34 ± 1.89	31.03 ± 0.97	12.30 ± 0.64	0.85 ± 0.04	0.42 ± 0.02	Throughout the year	Green
Malaysia	142.16 ± 3.32	34.17 ± 0.94	13.41 ± 0.49	0.79 ± 0.03	0.49 ± 0.01	Throughout the year	Green

## Data Availability

All the relevant data are provided along with the manuscript as Supplementary Files.

## Supplementary Materials

The following are available online at https://www.mdpi.com/article/10.3390/biom11060771/s1, Figure S1: Floral organ development and morphogenesis of A. graminifolia, Figure S2: Comparison of qRT-PCR expression intensities of selected TFs (a) with that of transcriptomic expression as FPKM (b), Table S1: Individual ratio (%) of annotation of DEGs related to flowering and hormonal regulation, Table S2: Gene IDs and FPKM values of the TFs used to filter stage-specific TFs, Table S3: Similarity index of some of the important flowering related TFs in Arundina graminifolia with other orchids and angiosperms.
