# Supplementary material for: Transcriptional Cascade in the Regulation of Flowering in the Bamboo Orchid Arundina graminifolia
Source: Biomolecules. 2021 May 21;11(6):771. doi: 10.3390/biom11060771 (PMC8224086; doi:10.3390/biom11060771)
Supplement: Supplementary file 1 [file biomolecules-11-00771-s001.zip › biomolecules-1188301-supplementary.pdf]

Supplementary Materials

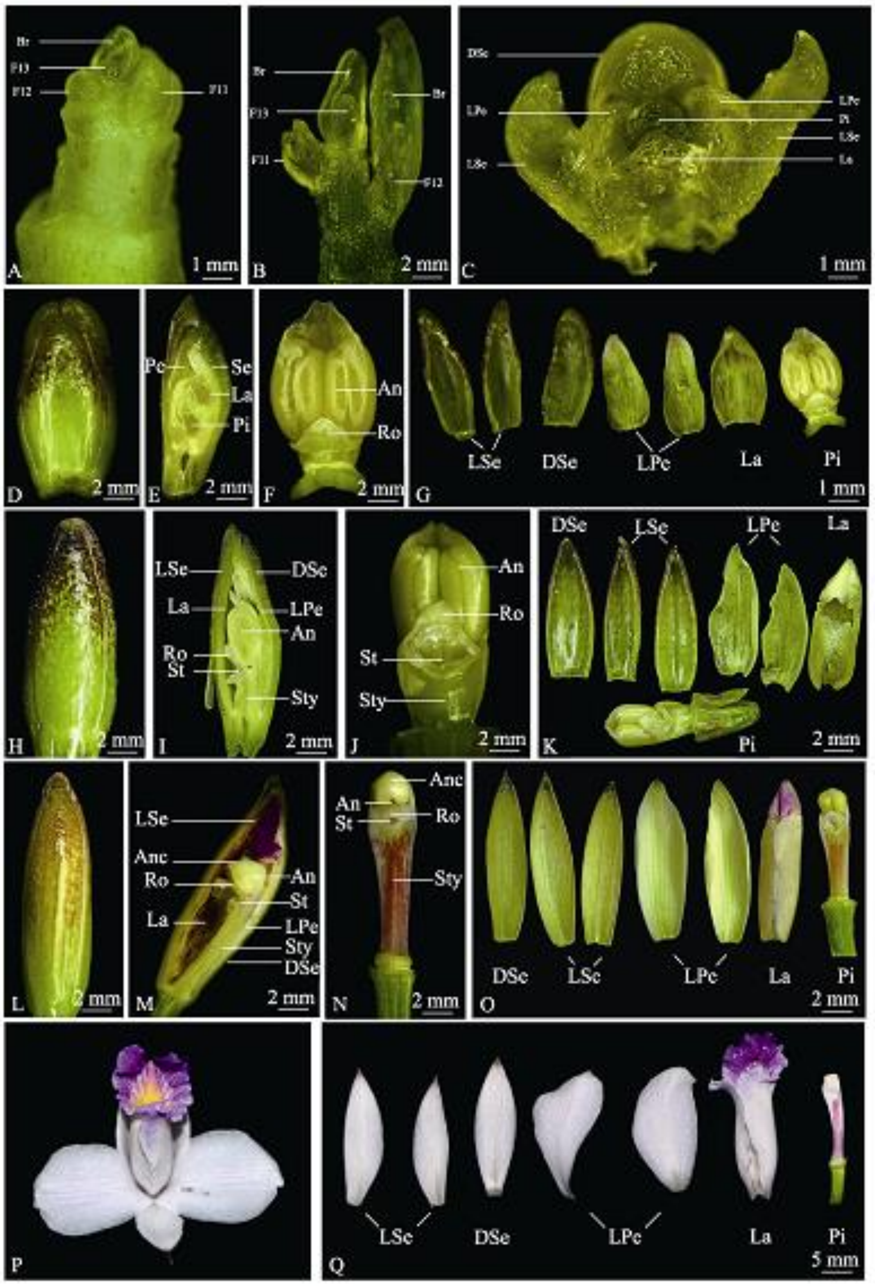

Supplementary Figure S1. Floral organ development and morphogenesis of *A. graminifolia*. **A–C**: Flower bud differentiation period 1; **D–G**: Sepal growth period 2; H-K: Pillar development period 3; **L–O**: Flower dyeing period 4; **P–Q**: Flowering period 5; **A**: The inflorescence; **B**: The inflorescence longitudinal section; **C**: Flower 1 (Fl 1); **D**, **H**, **L**: The whole flower bud; **E**, **I**, **M**: The longitudinal section of the flower bud; F, J, N: The pillar; **G**, **K**, **O**, **Q**: The organ anatomy; **P**: The flower; Se: Sepal; LSe: The lateral sepal; DSe: The dosal sepal; LPe: Lateral petal; La: Labe llum; Pi: Pillar; Br: Bract; Fl 1, Fl 2, Fl 3: Flower 1, F lower 2, Flower 3; An: Anther; Ro: Rostellum; St: Stigma; Sty: Style; Anc: Anther cap.

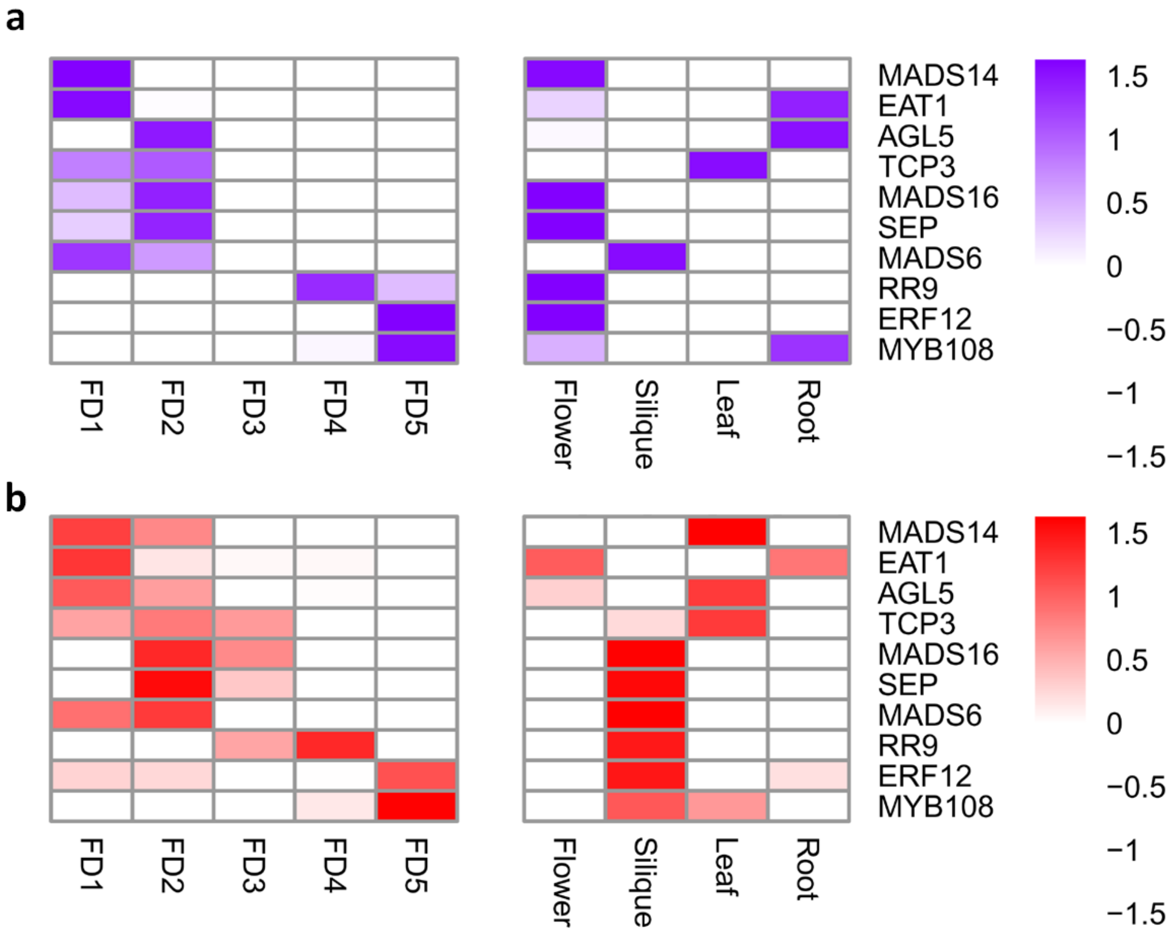

Supplementary Figure S2. Comparison of qRT-PCR expression intensities of selected TFs (a) with that of transcriptomic expression as FPKM (b).

Supplementary Table S1. Individual ratio (%) of annotation of DEGs related to flowering and hormonal regulation.

DB	Ratio (%)
GO	67.73
KEGG	36.42
Pfam	64.18
SwissProt	56.54
eggNOG	74.37
NR	72.29
